# Effect of Functionalised and Non-Functionalised Carbon Nanotubes-Urea Fertilizer on the Growth of Paddy

**DOI:** 10.21315/tlsr2018.29.1.2

**Published:** 2018-03-02

**Authors:** Norazlina Mohamad Yatim, Azizah Shaaban, Mohd Fairuz Dimin, Faridah Yusof, Jeefferie Abd Razak

**Affiliations:** 1 Faculty of Manufacturing Engineering, Universiti Teknikal Malaysia Melaka, Hang Tuah Jaya, 76100 Durian Tunggal, Melaka, Malaysia; 2 Department of Biotechnology Engineering, Kulliyah of Engineering, International Islamic University Malaysia, P.O. Box 10, 50728 Kuala Lumpur, Malaysia; 3 Carbon Research Technology Research Group, Engineering Materials Department, Faculty of Manufacturing Engineering, Universiti Teknikal Malaysia Melaka, Hang Tuah Jaya, 76100, Durian Tunggal, Melaka, Malaysia

**Keywords:** Functionalised, Multi-walled Carbon Nanotubes, Urea Fertilizer, Paddy

## Abstract

The roles of multi-walled carbon nanotubes (MWNTs) and functionalised multiwalled carbon nanotubes (fMWNTs) in enhancing the efficacy of urea fertilizer (UF) as plant nutrition for local MR219 paddy variety was investigated. The MWNTs and fMWNTs were grafted onto UF to produce UF-MWNTs fertilizer with three different conditions, coded as FMU1 (0.6 wt. % fMWNTs), FMU2 (0.1 wt. % fMWNTs) and MU (0.6 wt. % MWNTs. The batches of MR219 paddy were systematically grown in accordance to the general practice performed by the Malaysian Agricultural Research and Development Institute (MARDI). The procedure was conducted using a pot under exposure to natural light at three different fertilization times; after a certain number of days of sowing (DAS) at 14, 35 and 55 days. Interestingly, it was found that the crop growth of plants treated with FMU1 and FMU2 significantly increased by 22.6% and 38.5% compared to plants with MU addition. Also, paddy treated with FMU1 produced 21.4% higher number of panicles and 35% more grain yield than MU while paddy treated with FMU2 gave 28.6% more number of panicles and 36% higher grain yield than MU, which implies the advantage of fMWNTs over MWNTs to be combined with UF as plant nutrition. The chemical composition and morphology of UF-MWNTs fertilizers which is further characterised by FTiR and FESEM confirmed the successful and homogeneous grafting of UF onto the fMWNTs.

## Abstract (ru)

Peranan tabung karbon nano berdinding banyak (MWNTs) dan *functionalised* tabung karbon nano berdinding banyak (fMWNTs) dalam meningkatkan keberkesanan baja urea (UF) untuk pertumbuhan MR219 padi tempatan diselidiki. MWNTs dan fMWNTs telah digabungkan dengan UF untuk menghasilkan baja urea-tabung karbon nano berdinding banyak (UF-MWNTs) dengan tiga keadaan yang berbeza, dikodkan sebagai FMU1 (0.6 wt.% fMWNTs), FMU2 (0.1 wt.% fMWNTs) dan MU (0.6 wt.% MWNTs. Padi MR219 telah ditanam secara sistematik mengikut prosedur yang dilakukan oleh Institut Penyelidikan dan Kemajuan Pertanian Malaysia (MARDI). Prosedur ini dijalankan di dalam pot di bawah pendedahan cahaya matahari secara semula jadi dengan teknik pembajaan sebanyak tiga kali, selepas tabur (HLT) iaitu pada 14, 35 dan 55 hari. Menariknya, didapati bahawa pertumbuhan padi dengan pembajaan FMU1 dan FMU2 telah meningkat dengan ketara sebanyak 22.6% dan 38.5% berbanding pertumbuhan padi dengan pembajaan MU. Juga, padi yang dibaja dengan FMU 1 menghasilkan 21.4% bilangan tangkai yang lebih tinggi dan hasil bijirin 35% lebih tinggi daripada MU, manakala padi yang dibaja dengan FMU 2 memberikan nilai 28.6% dan 36% lebih tinggi jumlah tangkai dan hasil bijirin daripada MU. Ini telah menunjukkan kelebihan fMWNTs ke atas MWNTs untuk digabungkan dengan UF sebagai baja. Komposisi kimia dan kajian morfologi UF-MWNTs yang telah dicirikan oleh FTIR dan FESEM menunjukkan kejayaan dan kesebatian campuran gabungan UF dengan fMWNTs.

## INTRODUCTION

The landscape of modern technology in agriculture has been reshaped through recent development in nanomaterials (NMs) as a smart delivery system for efficient plant growth. NMs as smart delivery systems are well documented in the field of medicine, particularly for drugs and gene delivery (Panyam & Labhasetwar 2003; Kam *et al.* 2005; Zharov *et al.* 2005; Roco 2003; Lu *et al.* 2008). In agriculture, research on smart delivery systems are reported on the delivery of pesticides encapsulated in NMs for UV-shielding (Li *et al.* 2007) and assisted delivery of genetic material for crop improvement (Vijayakumar *et al.* 2010). Ghormade *et al.* (2011) and Wilson *et al.* (2008) have shown potential applications of NMs in agriculture to reduce the use of fertilizers by assisting in the controlled and slow-release of fertilizers. The NMs smart delivery system has led to the advancement of agriculture technology due to unique properties such as time control, specific targets, highly controlled, remotely regulated/pre-programmed/self-regulated and multifunctional characteristics avoiding biological barriers for effective targeting (Nair *et al.* 2010). In addition, the application of NMs reduces fertilizer use and increases agriculture yields through an optimized nutrient management (Srilatha 2011; Bhattacharyya *et al.* 2010; Garcia *et al.* 2010; Rashidi & Khosravi-Darani 2011; Sharon *et al.* 2010). NMs delivery system targets the plant to take up nutrients efficiently and enhance the germination rate of plants by improving the intake of water as well as oxygen (Zheng *et al.* 2005; Khot *et al.* 2012). Hence, leaching and losses of nutrients to unintended targets like soil are reduced.

Owing to MWNTs special physicochemical properties, which is the unique ability as molecular transporter for plant cells walls (Liu *et al.* 2009) that stimulates crop growth, improves the soil environment and promotes crop growth metabolism (Lu *et al.* 2002; Liu *et al.* 2007; Xiao *et al*. 2008). This ability makes MWNTs a great promise for progress in agricultural activities. The findings of the research on the impacts of carbon-based NMs combined with fertilizer on plants was reported by Liu *et al.* (2008) indicating the saving of nitrogenous fertilizer for winter wheat. Besides, they also found an increase of the yield and quality of the crop. A study of the effects of carbon NMs fertilizer on late rice by Qian et al. (2010) in the double rice season area of Southern China revealed that the use of carbon NMs fertilizer increases the number of glume flowers per year, fertility and the rice yield. Concurrently, the carbon NMs fertilizer was observed to slow down the rate of fertilizer release, hence reducing the amount of fertilizer used and improving the nitrogen uptake efficiency (NUE). This positive result agreed well with the role of carbon NMs in fertilizer for the enhancement of plant growth and yield. It encouraged researchers to explore the potential of combined carbon NMs with fertilizers in agriculture.

These findings are important in improving nitrogenous fertilizers which are mainly contributed by the urea fertilizer (UF) efficiency especially for paddy growth. Nitrogen (N) plays a critical role in paddy growth and productivity as it is required for the synthesis of many essential molecules including nucleic acids (DNA and RNA), amino acids and proteins (Fan *et al.* 2014; Lea & Jean-Francois 2001; Epstein 1972). Referring to the International Fertilizer Industry Association’s (IFA) Fertilizer Outlook 2013–2017, global N fertilizer demand would grow slightly to 107.5 million metric tons of N. The data presented showed a large volume of fertilizers used by the agricultural sector, especially the N fertilizer. However, it is not easy to increase the efficiency of mineral N fertilizers because plants normally take up N as nitrate or ammonium ions. It was reported that between 50% and 70% of the nitrogenous fertilizer is lost through leaching and gas emission of ammonia and nitrogen oxides to the atmosphere (Maene 1995). These may contribute to unfavourable environmental impacts and higher operational costs to farmers. Uncontrolled applications of UF also have adverse effects to aquatic life due to eutrophication causing excessive algae growth.

However, nanotoxicology of pristine MWNTs used with UF had raised great concerns regarding the side effects, biodegradability and their translocation into the food chain (Fiorito *et al.* 2006). Sayes et al. (2006) reported a high degree of CNTs functionalization would lead to a significant reduction in toxic effects, and this is important to eliminate cross contamination in the food chain during crop agricultural activities. Furthermore, functionalisation is important for further modification of MWNTs. Thus, functionalisation on MWNTs is essential in order to use them in the agricultural field. The main aim of this work is to study the different effects of pristine MWNTs and functionalised MWNTs combined with UF on paddy growth rate. In this study, we exposed the MR219 local paddy variety to MWNTs combined with UF in soil and observed the effects on their growth rate.

## MATERIALS AND METHODS

### Preparation of UF-MWNTs Fertilizer

Commercially available chemical vapour deposition-grown MWNTs with purity of >95% were used. The fMWNTs with 4 wt. % of carboxyl (-COOH) functional groups were purchased from Cahaya Tech, Malaysia. Various amounts of MWNTs and fMWNTs were sonicated before stirring with urea fertilizer for 6 hours at room temperature and 150 rpm. Samples were then dried in the oven at 70°C for 280 minutes.

### Plant Materials and Growth Conditions

The Malaysian local paddy variety (MR219) rice seeds were rinsed thoroughly and soaked for 24 hours in water. The seeds were then left to germinate on plastic containers for 24 hours. Uniform seedlings were selected and then transferred to differently labelled pots containing soil samples collected from agricultural field for wet rice *(Oryza sativa* L.) with field capacity of 32% and N content of 0.2%. All seedlings were grown for 110 days with variable UF-MWNTs fertilizer treatments. Ordinary fertilizers such as phosphorus (DAP) and potassium (muriate of potash) were applied in one time application at a rate of 50 kg ha^−1^. The paddy was grown in a pot under natural light conditions. The fertilizer was applied three times, that is, 14, 35 and 55 days after the sowing period at a rate of 120 kg ha^−1^ and following a standard procedure set by (MARDI). The experiments were performed for 110 days after sowing.

### Characterization of Fertilizer

Imaging was performed using a field emission scanning electron microscope (FESEM), Hitachi SU8000 for morphological observation on samples.

### Plant Growth Analysis

The height of the plants were recorded weekly after the first application of fertilizer and used to plot the growth trend. Fresh plants were harvested after 110 days, and the number of panicles and filled grains were recorded. The plants were dried in the oven at 100°C for 24 hours, and their weights were recorded as TDW. Consequently, they were grounded for Total N content analysis. Total nitrogen content was measured according to the Kjeldahl method (Li *et al.* 2008).

## RESULTS AND DISCUSSION

### Total Nitrogen (N) Evaluation

Total N content of different UF-MWNTs fertilizer (FMU1 with 0.6 wt. % fMWNTs, FMU2 with 0.1 wt. % fMWNTs, and MU with 0.6 MWNTs), that bind on the surface of MWNTs and paddy straw treated with different UF-MWNTs fertilizers were shown in Table 1. UF is used as the main fertilizer for paddy due to high value of N content which is 46%. This means that any process to enhance the efficiency of UF should maintain the N content. Obviously, total N content in MU was much lower than the original N content of UF, which was 44.8%. Fortunately, total N content of FMU1 and FMU2 were still within the acceptable limit, which was 45.3% and 45.1% respectively. This indicates that the amination process to introduce N from UF onto the surface of fMWNTs successfully prevented any losses of N from UF compared to the amination process with MWNTs. Functional groups on the surface of fMWNTs activated their surfaces active enabling them to retain more N during the amination process than non-active sidewalls of MWNTs.

**Figure 1:**
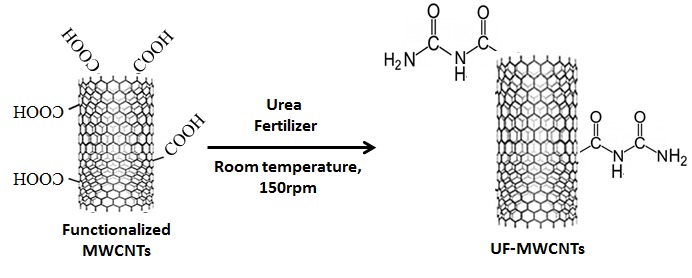
Scheme 1 shows amidation process.

After removing the excess UF through washing all the samples of UF-MWNTs fertilizer with deionised water, a small percentage of N content in UF were found to bind on the surface of fMWNTs. It has been discussed earlier that the advantages of MWNTs to have a unique ability as molecular transporter for walled plant cells is essential to promote crop growth and enhance soil environment. Thus, to realise this application, N from UF must be bound to MWNTs. Hence, further investigation on Total N content, which had successfully bounded on the surfaces of MWNTs and fMWNTs, was done and the data were shown in Table 1. MU gives the lowest bonded N content with only 0.28%, while FMU1 provides the highest N content that binds with the functional group on the surface of fMWNTs with 1.4%, followed by FMU2 with 1.27%. High amounts of fMWNTs (0.6 wt. %) will provide more functional groups and have wider active sites available for N to bind onto the surface of fMWNTs compared to low amounts of fMWNTs (0.1 wt. %) used. However, MU doesn’t have any functional groups provided on their surface, thus almost no N is available on their non-active surface after ensuing the removal process. Functionalisation or chemical modification of CNT—that is, the covalent attachment of carboxyl groups to the surface—has been used to introduce chemical specificity and process ability in different environments. This makes the sidewalls of MWNTs active for further amination processes to occur. The amination process on the surface of fMWNTs is described in scheme 1 of Fig. 1.

Treatment with different UF-MWNTs fertilizer also resulted in different total N content in the paddy straw harvested at 110 days after sowing. According to Table 1, paddy straw treated with MU showed higher N content at 1.7%. On the other hand, lower N content was recorded for the paddy straw treated with FMU1 and FMU2 at 0.93% and 0.99% respectively. On the contrary, high growth, TDW and grain yield were recorded for paddy applied with FMU1 and FMU2, which will be discussed later. Low N content of paddy straw by the end of the grain filling stage treated with FMU1 and FMU2 might imply better N utilization during this stage. Most plants take N from the soil continuously throughout their growth period, and N demand usually increases as plant size increased. Providing suitable N allows an annual crop such as paddy to grow to full maturity rather than delaying it. During the maturity stage—where the grain filling process are completed—about 40 percent of the N taken up by paddy will remain in their vegetative plant parts such as straw (Singh *et al.* 2013). Hence, low values of N remain in the straw indicating better utilisation of N for plant growth. This explains the low N content for paddy treated with FMU1 and FMU2. It was clearly observed that at the end of grain filling stage, the paddy treated with MU still had high N content remaining in the straw, thus suggesting low N utilisation for growth throughout their lives.

### Chemical Composition Identification using Fourier Transform Infrared Spectroscopy (FT-IR)

**Figure 2:**
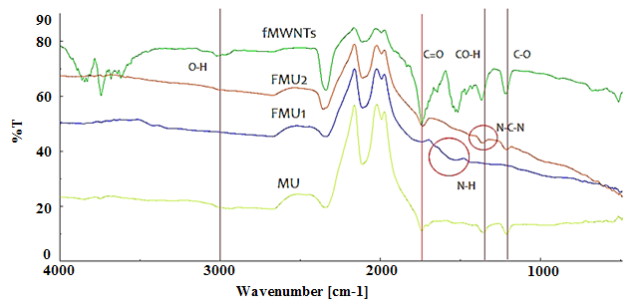
FTiR spectra of (a) fMWNTs, (b) FMU1, (c) FMU2 and (d) MU.

FT-IR aims to study the chemical composition of samples and the distribution of frequencies used to identify the presence of functional groups (Queiroz *et al.* 2003; Yu *et al.* 2004). Fig. 2 (a–d) shows the FT-IR spectra of fMWNTs, FMU1, FMU2, and MU. The outer walls of MWNTs are chemically inactive and can be activated through the chemical functionalisation process. Chemical functionalisation is based on the production of covalent bonds between the functional groups with the carbon of MWNTs (Jeon *et al.* 2011). It can be performed at the end caps of nanotubes or at their sidewalls, which may have many defects. Hence, upon reactions between UF and fMWNTs, it is strongly suggested that covalent bonding had occurred between the NH_2_ groups of UF and the carboxyl groups of fMWNTs.

The spectra mainly characterised by bands at 3300-2500 (O-H stretching vibration), 1700–1725 cm^−1^ (C=O stretching vibration) and 1210–1320 cm^−1^ (C-O stretching vibration) correspond to the vibration of the carboxylic acid groups (Wei *et al*. 2010). In the IR spectra of UF-MWNTs, the bands around 1640–1690 cm^−1^, 1510–1600 cm^−1^, 1376–1388 cm^−1^ and 1120–1290 cm^−1^ could be assigned to C=O stretching vibration, N-H bending, CH_2_ bending and C-N stretching. These characteristic spectral are attributed to amide (Wei *et al.* 2010).

Any changes in peak position or shape reveal that a change has happened in the distribution of frequencies in that particular vibration mode. By comparing the FT-IR spectra of MWNTs, fMWNTs and UF-MWNTs, it could be seen that the transmission peak of O-H stretching vibration at 3018 in the IR spectrum of fMWNTs (Fig. 2(a)) disappeared in the IR spectrum of UF-MWNTs (Fig. 2(b) and Fig. 2(c)), indicating that the O-H groups in fMWNTs might react with the amino groups in the urea fertilizer during the functionalisation process. Also, C=O (1739 cm^−1^) and C-O (1219 cm^−1^) stretching vibration attributed to fMWNTs were observed to have been replaced with new shapes of transmission peaks that appear, to correspond to N-H bending and C-N stretching vibration at 1519 and 1219 cm^−1^ respectively, after fMWNTs was further functionalised with UF. The NH_2_ groups from UF can react with the carboxyl groups (COOH) on the surface of fMWNTs and produce amide groups (Gao *et al.* 2005). Thus, the results from the FTiR spectra confirmed that chemical functionalisation has occurred between fMWNTs and UF instead of physical functionalisation (non-covalent) only.

The results obtained supported the Total N content data reported earlier, which revealed that a small percentage of N are bonded on to the surface of fMWNTs for FMU1 and FMU2 samples.

### Crop Growth

**Figure 3:**
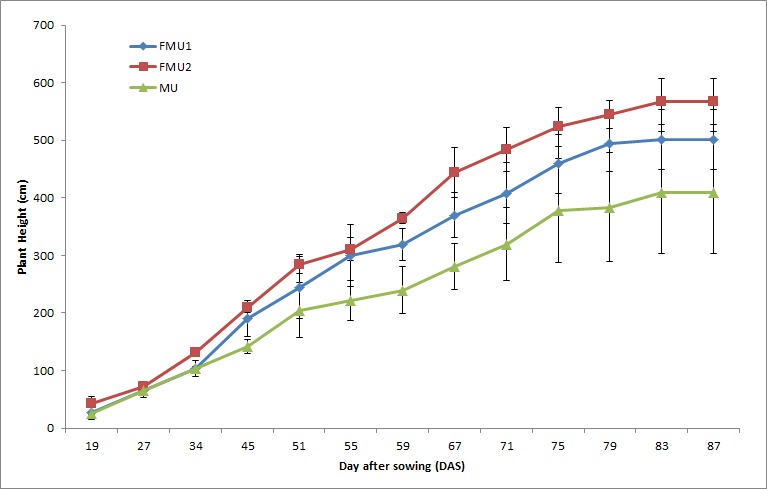
Crop growth trend in different UF-MWNTs fertilizer.

The trend of crop growth for different UF-MWNTs fertilizers was illustrated in Fig. 3. There were significant differences between the three types of UF-MWNTs fertilizers (FMU1, FMU2, and MU) above 45 DAS. Crop growth increased to the maximum until the flowering stage (83 DAS) and maintained constancy at the grain filling and physiological maturity (PM) stage. Finally, the grains were harvested at the PM stage when the grains were firmed and turning brown, two weeks before the full maturity stage. The crop growth percentage of paddy supplied with FMU1 and FMU2 was higher than MU by 38.5% and 22.6% respectively. The results might signify that fMWNTs in FMU1 and FMU2, enables N from UF to be absorbed efficiently by the plant and lead to a better growth rate. The results agreed with the explanation by Ghormade *et al*. (2011) on the interaction between plant cells and the fMWNTs that leads to the modification of the plant gene expression as well as associated biological pathways which increase N absorption and utilization by plants. This eventually enhances the plant growth and developments because efficient utilization of nitrogenous fertilizer by paddy is essential due to the role of N in the cell division (Marschner 1988; Timothy & Joe 2003). Thus, an efficient nitrogenous fertilizer including UF will significantly increase growth rate of paddy due to sufficient N supply.

Meanwhile, the low crop growth observed for paddy treated with MU can be explained by the toxicity properties of pristine MWNTs when interacting with plant cells. They will lead to plant cell apoptosis and increases the response oxidative stress (ROS), which results in cell viability reduction and cell death (Tan *et al.* 2009). This kind of toxicity effects might correlate with aggregation formations of pristine MWNTs (De La Torre-Roche *et al.* 2013) suspended in UF. Researchers claim that the paddy cells in the suspensions were found to demonstrate a self-defence response when exposed to pristine MWNTs, by sacrificing a small population of cells that aggregates with the MWNTs and precipitates (Tan & Fugetsu 2007; Rico *et al.* 2011). Similarly, Shen *et al.* (2010) also reported on the induction of a self-defence response through apoptosis in rice cells by SWNTs. This will eventually delay the plant growth.

Hence, fMWNTs revealed an advantage over pristine MWNTs that increases the paddy growth due to their unique and non-toxic properties which then increases the efficiency of UF and prevent cell death. This is important to eliminate cross contamination in the food chain when MWNTs are applied as plant nutrition.

### Total Dry Weight (TDW)

**Figure 4:**
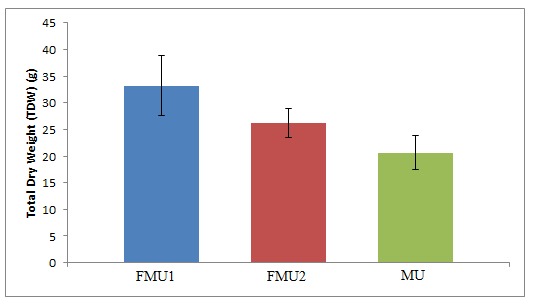
Total Dry Weight of paddy for three types (FMU1, FMU2 and MU) of UF-MWN Ts fertilizer.

There were significant differences in the values of TDW for FMU1, FMU2 and MU as shown in Fig. 4. The value of TDW for FMU1 and FMU2 increased by 61.2% and 27.2%, respectively compared with the value for MU. The TDW and crop growth data shows good correlation, which shows the advantage of fMWNTs over pristine MWNTs to be incorporated with UF as plant nutrition. Since both TDW and CGR data were associated with N content in plants (Fageria & Baligar 2007), it is strongly suggested that efficient absorption and utilization of N occurred through the application of fMWNTs in UF. The correlation of N content with photosynthetic activity was widely discussed (Fritsch & Jung 1984; Thomas *et al.* 1978). Efficient absorption and utilisation of N will enhance the process of photosynthesis and eventually result in high TDW production because N is a vital part that builds chlorophylls that absorbs the sunlight and convert light energy into sugars that fuel plant growth. Interestingly, Wu (2013) reported increment in chlorophyll content for paddy with application of fertilizer incorporated with carbon NMs.

Besides, the photosynthesis might also be enhanced due to the unique mobile and electrical properties of MWNTs in FMU, which have the potential to translocate from the soil into the plants and localize within the leaf cells (Giraldo *et al.* 2014). Giraldo *et al.* (2014) showed that MWNTs can passively transport and irreversibly localize within the plant chloroplasts and promote photosynthetic activity over three times higher than that of controls due to enhancement of maximum electron transport rates between MWNTs and chloroplasts. A few studies have demonstrated electron transfers between CNTs and photosynthetic machinery (Boghossian *et al.* 2011). The assembly of MWNTs within photosynthetic machinery was strongly suggested to modify the chloroplast activity of carbon capturing by promoting chloroplast solar energy harnessing and electron transport rates. Translocation capabilities of fMWNTs into the plant cells had been revealed in a few studies (Serag *et al.* 2011; Chen & Yada 2011). It is highly probable that hydrophilic, covalently functionalised and short fMWNTs give them an advantage to transport into the plant cells as compared to hydrophobic, pristine, agglomerate and long MWNTs. Thus, the significantly higher values of TDW for paddy with FMU treatment as compared to MU treatment might be explained by the localization of MWNTs in the chloroplast, which improves the process of photosynthesis. Obviously, this observation reveals the importance of functionalisation on MWNTs surfaces in enhancing the interaction between UF-MWNTs and plant cells.

### Evaluation of Yield Components

The effects of different UF-MWNTs fertilizers on yield components of paddy, specifically the number of panicles and the grain yield are shown in Table 2. Referring to Table 2, again the paddy under FMU1 and FMU2 treatments contributed a higher number of panicles, which lead to higher grain yield compared to paddy under MU treatment. Specifically, FMU1 and FMU2 treatment produced 21.4% and 28.6% increase in the number of panicles and 35% and 36% higher grain yield than MU treatment, respectively. The application of fertilizer containing carbon NMs were reported elsewhere to increase the grain yields of paddy by more than 10% (Liu *et al.* 2008; Liu *et al.* 2009; Wu 2013). Similar to crop growth and TDW, higher levels of available N is the likely explanation for the high yield of components including the number of panicles and grain yield produced (Azarpour *et al.* 2014).

This observation was strongly correlated with efficient delivery and utilisation of N by paddy through application of fMWNTs in UF. The efficient delivery might be due to translocation of fMWNTs into the plant roots, providing new pores and enhancing the uptake of soil water, which is saturated with nutrients by plants (Ma *et al.* 2009). The movement of water into the root cells brings together nutrients including N in the soil. MWNTs were reported earlier to create new pores through direct penetration into the seed coat of tomato (Khodakovskaya *et al.* 2009). The penetration was claimed to support water uptake inside the seeds and enhance plant growth. MWNTs penetration into the plant cells to promotes water uptake was also addressed by Tripathi *et al.* (2011). Besides, fMWNTs could also enter the plant cells via endocytosis instead of penetration and preferentially localized within the cells (Liu *et al.* 2009; Yaron *et al.* 2011). These fMWNTs will possibly be incorporated within the xylem in the plant roots, acting with them to form several new capillaries that encourage the water uptake potential in the plant in addition to the natural flow. This concept which is known as “large capillary” formation could enhance the delivery of N in the plants through enhanced water uptake capabilities. The fMWNTs in individual tubes were suggested to be easily translocated into the root cells and form several new capillaries in the xylem to enhance delivery of N compared to bundles of pristine MWNTs. This explains the low yield production for paddy treated with MU and implies fMWNT as an emerging technology in agriculture that encourages the plants to absorb nutrients and water.

### Morphological Observation of Fertilizer using Field Emission Scanning Electron Microscopy (FESEM)

**Figure 5:**
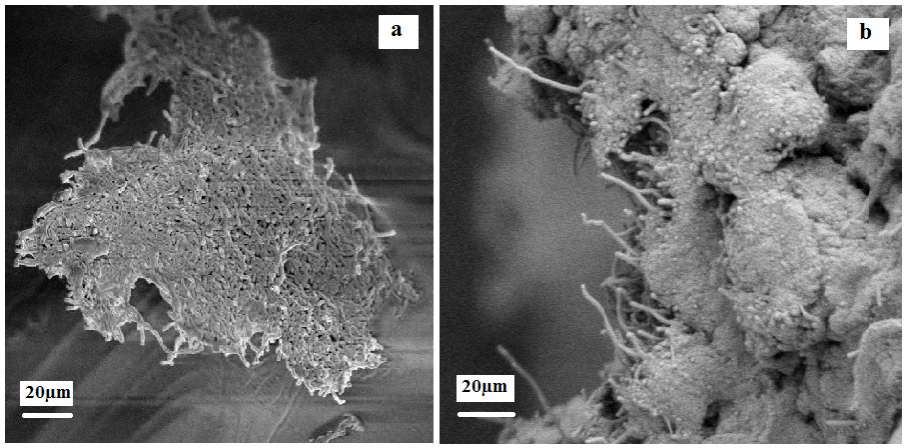
FESEM micrograph of (a) MU and (b) FMU.

In order to investigate the interaction of pristine and functionalised MWNTs with UF, FESEM images were captured to reveal their morphology properties. Fig. 5 illustrates the FESEM images of the distribution of fMWNTs and MWNTs FMU1 and MU, respectively. There was a strong correlation between the resulting properties of produced UF-MWNTs fertilizer with the morphological characteristics of the samples. The tubular structure of both MWNTs and fMWNTs in MU and FMU1 respectively (Fig. 5) are clearly shown. However, fMWNTs in FMU1 were found to be shorter due to the functionalisation process which results in erosion of their structure and shortening of the MWNTs (Wang *et al.* 2009). This observation supports a high percentage of crop growth, TDW and yield production observed for paddy treated with fMWNTs in FMU because shorter MWNTs had a stronger ability to penetrate protoplast plasma membranes of plant cells (Serag *et al.* 2011) and improve delivery of N as well as alleviate the toxicity observed for long CNTs (Ali-Boucetta *et al.* 2013).

The formation of pristine MWNTs and fMWNTs in UF were significantly different. Referring to Fig. 5(a), it is clearly observed that pristine MWNTs agglomerated with each other among the UF particles, which implies only physical interaction occurred between them and produced a non-homogeneous mixture of UF-MWNTs fertilizer. This observation was supported by the FT-IR results discussed earlier. Owing to their geometry and hydrophobic surface, pristine MWNTs have a tendency to form agglomerates with a bundle-like form in aqueous media (Wick *et al.* 2007). Cheng and Cheng (2005) addressed the accumulation properties of pristine MWNTs in aqueous environments where they tend to clump together and form aggregates in the micrometre range. MWNTs dispersed in a solvent fluid (UF solution) will rotate according to the Brownian motion theory. This rotation creates a large effective hydrodynamic radius and each nanotube sweeps out a spherical volume. Then, MWNTs will interact with each other’s hydrodynamic radius and result in entanglement and the creation of large agglomerates.

Surprisingly, researchers found that these aggregates did not change in size distribution with increasing salinity and temperatures that make them retain existence in the soil. This implies the correlation between the tendency to agglomerate and toxicity of MWNTs as have been widely reported (Wick *et al.* 2007; Poland *et al.* 2008; Patlolla *et al.* 2010). Pristine MWNTs which clump together and form aggregates were considered a biologically unfavourable form because when they reach living cells, they cause cell apoptosis and increased response oxidative stress (ROS), which can result in cell viability reduction and cell death even in low concentrations (Tan *et al.* 2009). Therefore, the toxicity issue has been alarming for the usage of pristine MWNTs in agriculture, especially when incorporated in fertilizers.

FMU1 in Fig 5(b) were observed to separate into individual tubes and homogeneously dispersed into UF. Here, functionalisation proved to eliminate aggregate formations of the fMWNTs successfully due to ionisable group productions on their sidewall. The carboxyl group, COOH, loses its hydrogen and becomes COO- when suspended in UF solution during preparation of samples. The repulsive forces between COO- groups will hold the nanotubes apart and prevent agglomeration. In agreement, Helland *et al.* (2007) reported in their review on environmental and human health knowledge of CNTs that MWNTs formed stable aggregates whereas fMWNTs showed a great dispersion variability due to looser structures and separation nanotubes at large-length scales. This clarifies significant reduction in toxic effects reported for high degree of CNTs functionalisation and promotes safer usage than pristine MWNTs.

**Figure 6:**
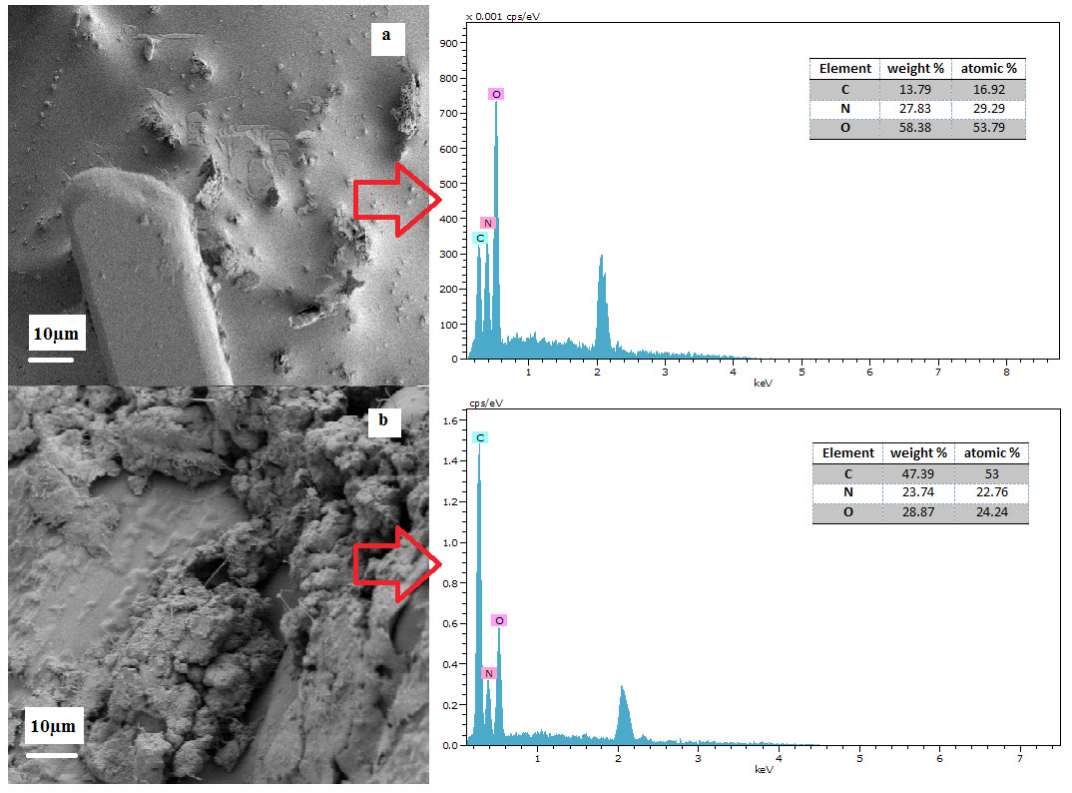
FESEM micrograph with EDX analysis of (a) MU, and (b) FMU.

FESEM images of UF-MWNTs with EDX analysis are depicted in Fig. 6. The EDX analysis of the area highlighted in Fig. 6(a) for MU revealed low carbon content. This indicates the absence of homogeneous pristine MWNTs in UF. This result supports earlier arguments which revealed that non- homogeneous pristine MWNTs in UF were due to accumulation. However, the increasing content of carbon from 16.92 atomic% of MU to 53 atomic% of FMU1 (Fig. 6(b)) can be clearly observed through EDX analysis, which shows good distribution of fMWNTs in the UF. Elements N and O were indication of UF. These are essential findings regarding pristine MWNTs and fMWNTs properties for further application in agriculture especially to be incorporated with fertilizers. The observations from FESEM and EDX analysis strongly suggest a good interaction between fMWNTs and UF as compared to pristine MWNTs.

## CONCLUSION

In this article, our data demonstrate essential growth characteristics for paddy treated with different UF-MWNTs fertilizers. The use of functionalised MWNTs (fMWNTs) in UF shows significant higher growth, yield components (number of panicles and grain yield) and TDW for paddy compared to pristine MWNTs. Total N content of paddy straw at PM stage was recorded to be lower for UF combined with fMWNTs than pristine MWNTs, indicating good N absorption and utilisation for producing high grain yield and high TDW. The advantages of fMWNTs over pristine MWNTs for plant nutrition were supported by FESEM images which show homogeneous and non-agglomerate fMWNTs in UF. Meanwhile, pristine MWNTs showed toxicity properties as they agglomerate with each other when combined with UF.

## Figures and Tables

**Table 1:** Total N content of different UF-MWNTs fertilizer and paddy straw treated with different UF-MWNTs.

UF-MWNTs fertilizer	Total N (%) of UF-MWNTs fertilizer	Total N (%) bind on the surface of MWNTs	Total N (%) of paddy straw
FMU 1	45.3	1.40	0.93
FMU 2	45.1	1.27	0.99
MU	44.8	0.28	1.7

**Table 2:** Number of panicles and grain yield of paddy for different UF-MWNTs fertilizer.

UF-MWNTs Fertilizer	Number of panicles	Grain yield
FMU 1	17	743
FMU 2	18	748
MU	14	550
